# Early prediction of unplanned critical care transfers in children using EHR-based ensemble machine learning

**DOI:** 10.1093/jamiaopen/ooag105

**Published:** 2026-06-13

**Authors:** Eamonn Tweedy, Sanjiv Mehta, Mark V Mai, Victor Ruiz, Helen Lingyun Shi, Gerald P Shaeffer, Akira Nishisaki, Naveen Muthu, Fuchiang (Rich) Tsui

**Affiliations:** Tsui Laboratory, Department of Biomedical and Health Informatics, Children’s Hospital of Philadelphia, Philadelphia, PA 19146, United States; Division of Critical Care Medicine, Department of Anesthesiology and Critical Care Medicine, Children’s Hospital of Philadelphia, Philadelphia, PA 19104, United States; Department of Anesthesiology and Critical Care Medicine, University of Pennsylvania, Philadelphia, PA 19104, United States; Department of Biomedical and Health Informatics, Children’s Hospital of Philadelphia, Philadelphia, PA 19146, United States; Department of Pediatrics, Emory University School of Medicine, Atlanta, GA 30322, United States; Information Systems and Technology, Children’s Healthcare of Atlanta, Atlanta, GA 30329, United States; Tsui Laboratory, Department of Biomedical and Health Informatics, Children’s Hospital of Philadelphia, Philadelphia, PA 19146, United States; Tsui Laboratory, Department of Biomedical and Health Informatics, Children’s Hospital of Philadelphia, Philadelphia, PA 19146, United States; Department of Biomedical and Health Informatics, Children’s Hospital of Philadelphia, Philadelphia, PA 19146, United States; Division of Critical Care Medicine, Department of Anesthesiology and Critical Care Medicine, Children’s Hospital of Philadelphia, Philadelphia, PA 19104, United States; Department of Anesthesiology and Critical Care Medicine, University of Pennsylvania, Philadelphia, PA 19104, United States; Department of Pediatrics, Emory University School of Medicine, Atlanta, GA 30322, United States; Information Systems and Technology, Children’s Healthcare of Atlanta, Atlanta, GA 30329, United States; Tsui Laboratory, Department of Biomedical and Health Informatics, Children’s Hospital of Philadelphia, Philadelphia, PA 19146, United States; Department of Anesthesiology and Critical Care Medicine, University of Pennsylvania, Philadelphia, PA 19104, United States; Department of Biomedical and Health Informatics, Children’s Hospital of Philadelphia, Philadelphia, PA 19146, United States; Department of Biostatistics, Epidemiology, and Informatics, University of Pennsylvania, Philadelphia, PA 19104, United States

## Abstract

**Background:**

Early recognition of unplanned ICU transfer is critical for improving outcomes, yet existing pediatric warning systems lack accuracy and timeliness. We developed machine learning models predicting unplanned ICU transfer up to 24 hours in advance.

**Methods:**

We conducted a retrospective cohort study using electronic health record data at a quaternary-care children’s hospital with 175 PICU/NICU beds. Patients aged 0-24 years admitted to medical or surgical wards for ≥30 hours between 2019 and 2024 were included. The cohort was divided into development (2019-2022) and external validation (2023-2024). The F-WIN model is an ensemble of extreme gradient-boosting models trained at varying prediction horizons (1-24 hours). The primary outcome was unplanned transfer to PICU/NICU, defined as transfer followed by clinical deterioration within 12 hours, including death, administration of vasopressors or inotropes, intubation, or new non-invasive ventilation. We compared F-WIN’s performance with the bedside Pediatric Early Warning System and the Watcher and Clinician Concern programs. Metrics included areas under the receiver operating characteristic (AUROC) and precision-recall curve (AUPRC).

**Results:**

Among 72 419 floor stays (49 283 patients) in the full dataset, 816 stays (1.1%) involving 769 patients (1.6%) ended in unplanned ICU transfer. Using 647 variables, F-WIN achieved strong discrimination across horizons (8-hour AUROC: 0.93; [95%CI, 0.91-0.95], AUPRC: 0.36 [95%CI, 0.29-0.43]); 24-hour AUROC: 0.91 ([95%CI, 0.89-0.93], AUPRC: 0.22 [95%CI, 0.17-0.29]) and outperformed all comparators. A parsimonious model preserved performance (24-hour *P* = .17; 8-hour *P* = .06).

**Conclusions:**

F-WIN accurately predicted unplanned transfer up to 24 hours in advance, outperforming existing tools. A parsimonious model maintained similar performance.

## Lay Summary

Identifying clinical deterioration in hospitalized children is a top safety priority, as delayed recognition can lead to preventable ICU transfers, increased costs, and higher mortality. Existing systems, including the bedside pediatric warning system (bedsidePEWS), fall short in accuracy or timeliness. To address this, we developed F-WIN (Floor-to-ICU Warning Index), a machine learning model designed to predict unplanned ICU transfers–transfers followed by critical deterioration—up to 24 hours in advance. Using data from over 50 000 pediatric hospital admissions, we developed a 647-variable model using electronic health records data, including vital signs, lab results, and nursing assessments. In an external validation group of over 22 000 admissions, F-WIN generated accurate predictions up to 24 hours in advance, significantly outperforming comparator systems. We also developed a version of the model using only 62 key variables that maintained similar performance, making it easier to integrate into hospital workflows. These results show that F-WIN can provide reliable predictions of unplanned ICU transfer 24 hours in advance. Finding a patient at risk earlier may allow care teams to coordinate safer, proactive transfers and interventions before the patient reaches a state of crisis, potentially reducing complications and improving outcomes for the most vulnerable hospitalized children.

## Background

Among the top 10 pediatric safety priorities identified by parents and health system leaders, improved recognition and response to clinical deterioration ranked highest.^1^ Despite advances, hospitalized children experience acute deterioration and emergency ICU transfers causing morbidity and mortality. Each deterioration event potentially adds $100 000 to the cost of hospitalization^2^ and review of events suggests that more than 40% of events may be preventable.^3^

Even with widespread use of pediatric early warning systems (PEWS), children experience potentially preventable ICU transfers resulting in morbidity and mortality. In the EPOCH trial,^4^ the only large, cluster randomized clinical trial of the implementation of bedsidePEWS,^5^ the intervention was associated with a decrease in critical deterioration events but not a decrease in mortality. The recently developed pediatric Calculated Assessment of Risk and Triage (pCART) model^6^^,^^7^ was designed to predict ward to ICU transfer up to 12 hours in advance based on age and a collection of vital signs and laboratory results selected by experts, and achieved an area under the receiver operating characteristic (AUROC) of 0.84. This model was developed to predict any ICU transfer, regardless of subsequent deterioration, reflecting subjective clinician judgment and environmental factors like ICU census or staffing.

Our overall objective was to develop an electronic health record (EHR) driven, accurate machine learning (ML) model targeting unplanned transfer to ICU followed by clearly defined clinical deterioration events (CDE), upto 24 hours in advance. By requiring evidence of deterioration post-transfer, this definition excludes patients transferred for other reasons, ensuring our model focuses on patients with genuine clinical decline. We chose a 24-hour prediction window in an effort to increase the opportunity for proactive, safer transfers involving structured handoffs during peak staffing hours, mitigating the elevated risks associated with after-hours deterioration^8^ and emergent transfers.^9^ Our study aimed to: (1) determine whether data-driven ensemble ML modeling utilizing routinely captured electronic health record (EHR) data accurately predicts transfer to the ICU followed by CDE up to 24 hours prior to the transfer, (2) compare the performance with several clinically-used prediction models, and (3) develop a parsimonious model which approximates this model performance with fewer EHR variables for near-future clinical integration.^10^

## Methods

We applied ML techniques to develop and validate a model predicting transfer from a medical or surgical floor to an ICU followed by a CDE. The Institutional Review Board at the Children’s Hospital of Philadelphia (CHOP) reviewed and determined this study to be exempt research (IRB 20-017837). This study follows the Transparent Reporting of a Multivariable Prediction Model for Individual Prognosis or Diagnosis plus Artificial Intelligence (TRIPOD+AI)^11^ and STrengthening the Reporting of OBservational studies in Epidemiology (STROBE)^12^ reporting guidelines.

### Setting and population

We conducted a retrospective study of patients admitted to the CHOP Philadelphia Campus medical or surgical ward units from 2019 to 2024. CHOP is a free-standing quaternary children’s hospital with approximately 600 inpatient beds and 36 000 annual admissions.

We included patients aged 0-24 years who had a contiguous floor stay of at least 30 hours in length on a CHOP medical or surgical floor unit between January 1, 2019, and December 31, 2024. Patients aged 22-24 were included to capture >99% of floor admissions, ensuring a sample representative of actual care. We included only the first-floor stay of length at least 30 hours within each hospital encounter, in order to guarantee at least 6 hours of baseline data availability at the 24-hour prediction horizon. We excluded stays ending in a transfer to a cardiac care unit (CCU) or infant transitional care unit (ITCU), an intermediate observational unit mainly for cardiac or former NICU patients. We excluded patients with an active do not resuscitate (DNR) order during the floor stay.

### Unplanned ICU transfer with clinical deterioration events

We defined the primary outcome to be unplanned ICU transfer with CDE, which includes any of the following events within the 12 hours following the transfer: death, administration of vasopressors or inotropes for supporting cardiovascular function through continuous IV infusion, intubation, or initiation of new noninvasive ventilation (NIV). Patients who died on the floor with active code status were included, using time of death as transfer time.

**Figure 1.**
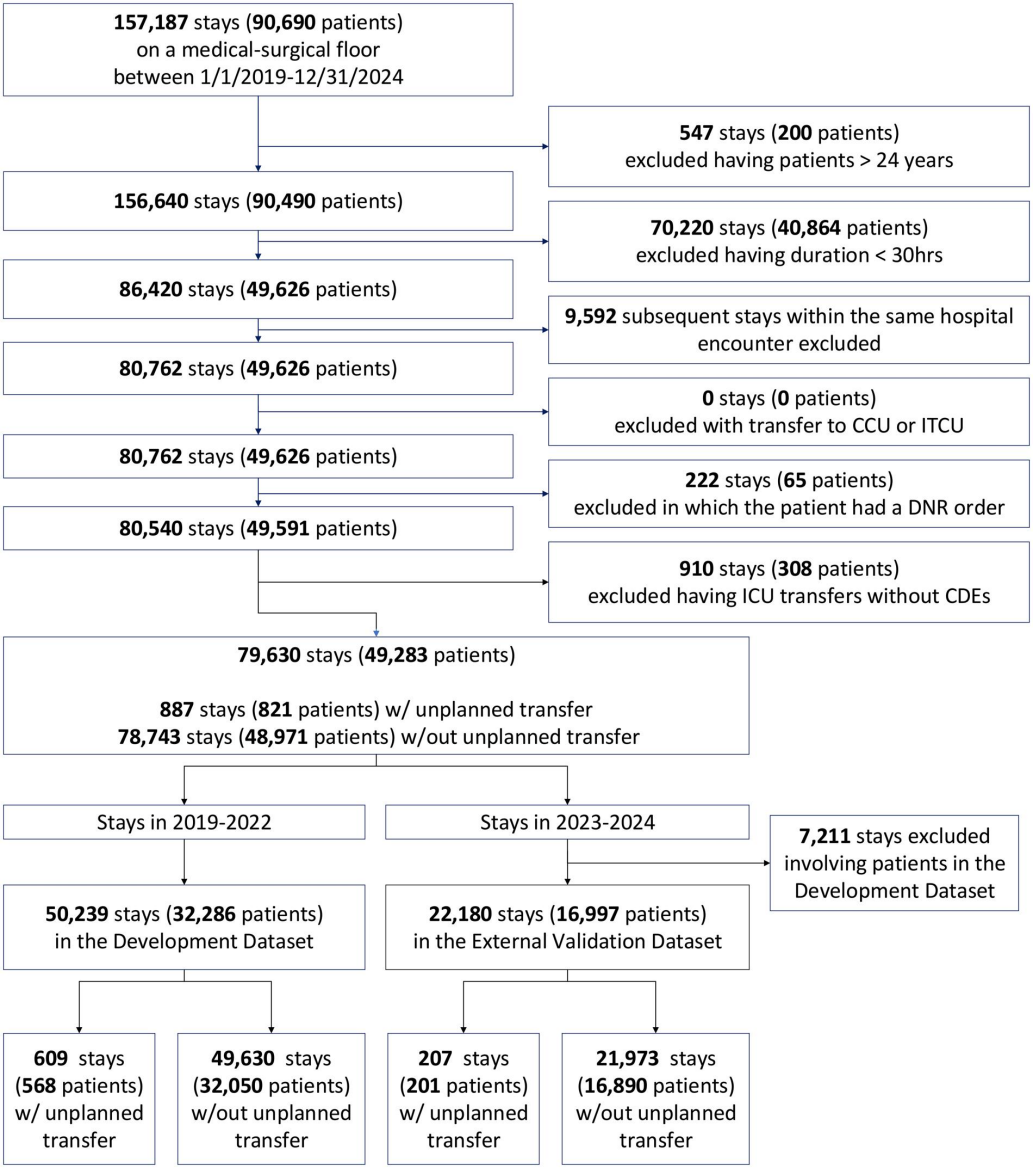
Flow chart of the cohort inclusion and exclusion process and the split into the development dataset and the external validation dataset. Abbreviations: CDE, Critical deterioration event; Unplanned transfer, transfer to a Pediatric ICU or Neonatal ICU unit followed by a CDE within 12 hours of transfer, or death on the floor; CCU, Cardiac Care Unit; ITCU, Infant Transitional Care Unit.

To develop our control cohort, for each floor stay not ending in an ICU transfer, we randomly sampled a “pseudo-transfer time,” excluding the first 30 hours of the floor stay. Restricting to stays at least 30 hours guarantees at least 6 hours of baseline data at the 24-hour horizon. Stays which ended in ICU transfer without subsequent CDE were excluded. Figure 1 shows the cohort inclusion and exclusion flowchart.

### Electronic health record and feature development

Information on patient stays, transfers, CDEs, and model inputs was obtained from the local electronic health record (EHR) (Epic Systems, Verona, WI, USA). Vasopressor and inotrope administration (epinephrine, dopamine, norepinephrine, dobutamine, phenylephrine, isoproterenol) was sourced from the medication administration record (MAR). We intentionally aligned our outcome definition with a prior definition for critical deterioration to preserve comparability with established work.^10^ In this context, vasopressin was excluded to avoid misclassifying patients receiving vasopressin for indications such as diabetes insipidus rather than for hemodynamic support, which could reduce the specificity of vasopressor use as a marker of clinical deterioration. Intubation was inferred from the earliest record of any of the following: a Line, Drain and Airway (LDA) entry for endotracheal tube, invasive ventilation documented in flowsheets, paralytic administration in MAR followed by invasive ventilation within 24 hours. NIV was identified by the earliest flowsheet entry or order.

Model variables were derived from structured EHR data, including demographics, diagnoses, medications, procedures, labs, vital signs, and nursing assessments; details are in the Supplemental document. Development incorporated 2172 medication, 1700 diagnosis, 368 procedure, 98 vital sign, 560 laboratory, 293 nursing assessment, and 4 demographic variables.

### Missing data handling

Demographic, medication, and diagnosis variables had no missing values. Missing laboratory, vital sign, and numerical nursing assessments were filled with training set medians. Missing categorical nursing assessment variables were accounted for by including a “missing” category.

### Development and external validation cohorts

We divided the cohort into: (1) a development dataset consisting of floor stays between 2019 and 2022, and (2) a temporally distinct external validation data set consisting of floor stays between 2023 and 2024. The external validation set excluded patients appearing in the development cohort.

### Ensemble machine learning model

We first developed an innovative ensemble of decision-tree-based eXtreme Gradient Boosting (XGB) models^13^ trained to predict the primary outcome at seven prediction horizons: 24, 12, 8, 6, 4, 2, and 1 hour prior to the transfer time or pseudo-transfer time. In the development cohort dataset, we used nested stratified cross-validation for training and hyperparameter optimization. The final ensemble prediction was an average of these seven predictions. The pipeline also ranked features by mutual information with the outcome and selected the top *k*, where *k* was a hyperparameter. Our best performing ensemble model will be referred to as the Floor-to-ICU Warning INdex (F-WIN) model.

Ensemble models were also developed using Random Forest^14^ (RF) and LASSO Logistic Regression.^15^ Additionally, we trained parsimonious models with feature selection fixed at *k* = 20, 40, and 60 variables, respectively.

### Existing clinically-used prediction models

We compared our best-performing model to three currently used clinical prediction models at each horizon. The Watcher program^16^ provides a clinician manually-entered flag indicating that a patient is deemed to be at high risk of deterioration. The Clinician Concern^17^ score is a manually-entered measure of how worried the care team is regarding the patient’s condition (0 = “No concern,” 1 = “Slight concern,” 2 = “Moderate concern,” 3 = “Significant concern”). The bedsidePEWS^5^ risk score, ranging from 0 to 26, is computed from 7 EHR elements from the prior 24 hours with age-specific references: heart rate, systolic blood pressure, capillary refill time, respiratory rate, respiratory effort, oxygen saturation, and oxygen therapy. We derived a binary prediction based on whether the patient was an active Watcher at prediction time, and numerical Clinician Concern and bedsidePEWS risk scores using the maximum score seen in the preceding 24 hours. BedsidePEWS and Watcher were available throughout 2023-024; Clinician Concern was sufficiently adopted only from July 2024, so we limited that comparison to July-December 2024. We also compared our model’s performance to that of binary predictions using clinically used thresholds–bedsidePEWS > 4 and Clinician Concern > 0. For these comparisons, we select F-WIN prediction thresholds which meet or exceed the specificity of the respective comparator.

### Model evaluation metrics

In the external validation cohort, we evaluated model performance, including the area under the receiver operating characteristic curve (AUROC), the area under the precision-recall curve (AUPRC), specificity, sensitivity, positive predictive value (PPV), F1-score, and the Brier Skill Score (BSS). We estimated 95% confidence intervals (CIs) for all metrics using 2000 stratified bootstrap replicates.^18^ For all model comparisons, we tested for statistically significant differences in model AUROC performance using Delong’s method.^19^ We tested differences in sensitivity for statistical significance using McNemar’s test,^20^ tested PPV differences using Kosinski’s weighted generalized score statistic,^21^ and tested F1-score differences using a two-sided permutation test with 10,000 iterates. All statistical comparisons were evaluated at a significance level of *P* < .05 corrected for multiple comparisons using false discovery rate control (Benjamini-Hochsberg Method^22^).

### Model fairness

We assessed the best-performing model and the best-performing parsimonious for variance across demographic factors including reported sex, race, ethnicity, payor, and age at the end of the observation window. Each between-group AUROC comparison was conducted using an unpaired Delong test.^19^

### Impact of model variables

We assessed the association between each feature and the outcome by computing adjusted odds ratios (AOR) in the development dataset. For simplicity, we limit this calculation to the set of features having nonzero feature importance in the best parsimonious model. Prior to computing AORs, we filtered variables on bivariate collinearity and mapped numerical variables to *Z*-scores.

We also assessed which features contribute most heavily to the model’s predictions on the external validation set using SHAP^23^ (SHapley Additive exPlanations) values, which measure the magnitude and directionality of each feature’s influence on the model’s predicted log-odds of the outcome.

### Secondary analysis including ICU transfers without CDE

We performed a secondary analysis in which we evaluated the performance of our best model on a modified version of the external validation cohort in which we included floor stays ending in ICU transfer without subsequent CDE, which had been excluded during model development and our primary model evaluation.

## Results

### Study cohort demographics

The development cohort (2019-2022) included 32 286 patients over 50 239 stays, of which 609 (1.2%) involved the primary outcome. The external validation cohort (2023-2024) included 16 997 patients over 22 180 stays, of which 207 (0.9%) involved the primary outcome. We excluded 7211 stays from the external validation cohort involving patients appearing in the development cohort. eTable 1 shows the breakdown of first CDE types among stays in the development and external validation datasets, respectively. The demographic details of patients in the development and external validation cohort, respectively, are summarized in Table 1. eTables 2 and 3 show the patient-level breakdown and univariate odds ratios of each demographic group based on whether they experienced the primary outcome for the development cohort and external validation cohort, respectively.

**Table 1.** Demographic characteristics of the development and external validation patient cohorts.

**Variable**	**Development cohort**	**External validation cohort**
**Number of patients**	32 286	16 997
**Sex, number of patients (%)**		
** Female**	16 010 (49.6)	8256 (48.6)
** Male**	16 273 (50.4)	8740 (51.4)
** Unknown**	3 (0.0)	1 (0.0)
**Race, number of patients (%)**		
** Black**	8735 (27.1)	3896 (22.9)
** White**	16 564 (51.3)	8741 (51.4)
** Other^a^**	6177 (19.1)	3649 (21.5)
** Unknown^b^**	810 (2.5)	711 (4.2)
**Hispanic of Latino Ethnicity, number of patients (%)**		
** Yes**	4224 (13.1)	2600 (15.3)
** No**	27 687 (85.8)	13 980 (82.2)
** Unknown^b^**	375 (1.2)	417 (2.5)
**Age, number of patients (%)**		
** < 28 days**	2327 (7.2)	1141 (6.7)
** >= 28 days, < 2 years**	7459 (23.1)	4178 (24.6)
** 2-11 years**	11 023 (34.1)	6064 (35.7)
** 12-18 years**	10 546 (32.7)	5285 (31.1)
** 19-24 years**	931 (2.9)	329 (1.9)
**Outcome^c^, number of patients (%)**		
** No floor stay ending in ICU transfer**	31 718 (98.2)	16 796 (98.8)
** At least one floor stay ending in ICU transfer followed by CDE**	568 (1.8)	201 (1.2)

Demographic fields from the EHR were collected during the first hospitalization for each patient in the development or external validation dataset, respectively.

a The “Other” race category contains patients whose self-reported race in the EHR was “Asian,” “Indian,” “Native Hawaiian or Other Pacific Islander,” “American Indian or Alaska Native,” or “Other.”

b The “Unknown” Race and Ethnicity categories comprise patients whose self-reported race in the EHR was “Choose note to disclose,” “Unknown,” “Asked but unknown,” or “Refused,” or who had multiple conflicting responses during the hospitalization.

c Patient-level outcomes are based on whether that patient experienced an ICU transfer followed by CDE during any stay contained in the development or external validation datasets, respectively.

### F-WIN model performance

Model type selection was based on cross-validation scores on the development dataset. Across the 7 horizons, the ensemble XGB model outperformed the RF and LASSO models with statistical significance, for example, AUROCs at the 24-hour prediction horizon: XGB 0.90 (95% CI, 0.89-0.91) vs LASSO 0.89 (95% CI, 0.88-0.91), *P* < .01 vs RF 0.89 (95% CI, 0.88-0.91), *P* < .01. eTable 4 summarizes the cross-validation performance of these models at all horizons. The ensemble XGB, henceforth called F-WIN, depends on a total of 647 variables The ensemble XGB model, referred to as F-WIN, uses 647 variables: 480 labs, 90 vital signs, 17 medications, 33 nursing assessments, 26 diagnoses, and 1 demographic variable. eTable 5 summarizes the cross-validation performance of the three parsimonious models at all horizons, among which the *k* = 60 model had the strongest performance. As each horizon model can depend on a different list of k variables, the ensemble models can depend on more than k variables. The resulting ensemble models fit on the entire development dataset depended on 29, 45, and 62 variables, respectively. The 62-variable parsimonious model will henceforth be referred to as the F-WIN parsimonious model.

F-WIN showed strong performance and good calibration across horizons. On the external validation dataset at the 24-hour prediction horizon, the F-WIN model achieved AUROC of 0.91 (95% CI, 0.89-0.93), AUPRC of 0.22 (95% CI, 0.17-0.29), and Brier Skill Score of 0.12 (0.09-0.16). At the 8-hour prediction horizons, the performance increased to an AUROC of 0.94 (95% CI, 0.92-0.95), AUPRC of 0.36 (95% CI, 0.29-0.43), and Brier Skill Score of 0.19 (0.16-0.23). The AUPRC of the F-WIN model was approximately 24 and 40 times the prevalence of 0.9% at the 24-hour and 8-hour horizons, respectively.

Choosing a prediction threshold to achieve specificity of 0.90 at the 24-hour horizon, F-WIN has sensitivity of 0.74 (95% CI, 0.68-0.80), PPV of 0.07 (95% CI, 0.06-0.07), and F1-score 0.12 (95% CI, 0.11-0.13) at the 24-hour horizon and specificity of 0.90 (95% CI, 0.89-0.90), sensitivity of 0.86 (95% CI, 0.81-0.91), PPV of 0.07 (95% CI, 0.07-0.08), and F1-score of 0.14 (95% CI, 0.13-0.14) at the 8-hour horizon. Increasing the threshold to achieve 24-hour specificity of 0.95 results in sensitivity of 0.58 (95% CI, 0.51-0.65), PPV of 0.10 (95% CI, 0.09-0.11), and F1-score of 0.17 (95% CI, 0.15-0.19) at the 24-hour horizon and specificity of 0.95 (95% CI, 0.95-0.96), sensitivity of 0.74 (95% CI, 0.68-0.80), PPV of 0.13 (95% CI, 0.12-0.14), and F1-score of 0.22 (95% CI, 0.20-0.24) at the 8-hour horizon. eTable 6 provides sensitivity, specificity, and PPV at a wider range of thresholds chosen to achieve target sensitivity or specificity values, and eFigure 1 shows plots of sensitivity, specificity, PPV, and F1-score across a full range of prediction thresholds, for the 24-hour, 12-hour, and 8-hour horizons.

Table 2 shows the AUROC and AUPRC performance of the F-WIN and F-WIN parsimonious models in the external validation dataset at 8, 12, and 24-hour prediction horizons, and eFigure 2 shows the ROC and PRC curves of these models. The full F-WIN model does not have statistically significantly better AUROC performance than the F-WIN parsimonious model on the external validation set at any of the 8, 12, or 24-hour horizons.

**Table 2.** Predictive performance of the F-WIN and Parsimonious F-WIN models and the bedsidePEWS risk score on the external validation dataset, at 24-hour, 12-hour, and 8-hour prediction horizons.

	24-Hour Horizon		12-Hour Horizon		8-Hour Horizon	
	AUROC	AUPRC	AUROC	AUPRC	AUROC	AUPRC
	(95% CI)	(95% CI)	(95% CI)	(95% CI)	(95% CI)	(95% CI)
**F-WIN model [*n* = 647 variables] (reference)**	0.91 (0.89-0.93)	0.22 (0.17-0.29)	0.92 (0.90-0.94)	0.28 (0.22-0.35)	0.93 (0.91-0.95)	0.36 (0.29-0.43)
**Parsimonious F-WIN model [*n* = 62 variables]**	0.90 (0.88-0.92)	0.19 (0.14-0.26)	0.92 (0.89-0.94)	0.24 (0.18-0.31)	0.93 (0.91-0.95)	0.30 (0.24-0.37)
**bedsidePEWS Score [*n* = 7 variables]**	0.76 (0.72-0.79)^a^	0.02 (0.02-0.03)	0.79 (0.75-0.81)^a^	0.03 (0.02-0.04)	0.80 (0.77-0.83)^a^	0.04 (0.03-0.04)

The prevalence of ICU transfer followed by CDE in the external validation dataset was 0.9%.

The bedsidePEWS Score consisted of the maximum bedsidePEWS entry seen in the 24-hour period prior to each prediction horizon.

a Statistically significant difference (Delong test *P* < .05) compared with the F-WIN model at the same prediction horizon.

F-WIN maintained strong performance on an expanded external validation dataset which included 241 stays ending in ICU transfer without CDE (24-hour AUROC: 0.91 [95% CI, 0.88-0.92], AUPRC: 0.18 [95% CI, 0.14-0.23]). At each horizon, the AUROC scores were not statistically significantly different from those on the main external validation dataset at a level of *P* < .05. eTable 7 shows the performance at all horizons.

### Impact of model variables

**Figure 2.**
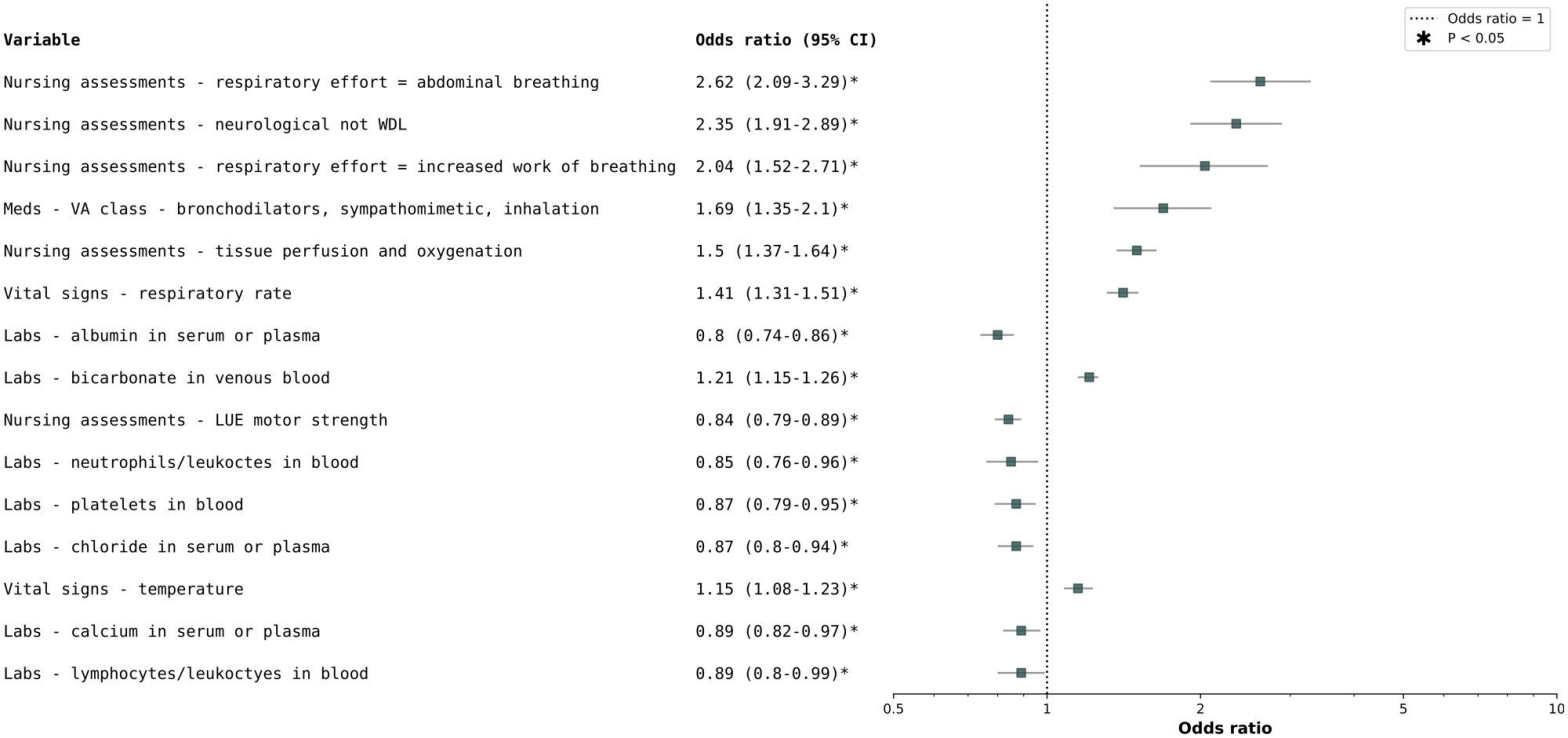
Adjusted odds ratio (AOR) forest plot showing the top 15 features with statistically significant odds ratios, ranked in descending order of absolute log-odds coefficient. The calculation was limited to features with nonzero feature importance in the 62-feature parsimonious F-WIN model, and features were screened in advance for bivariate collinearity. Nursing assessment and medication variables were binary variables. Labs and vitals were numerical variables, and were rescaled to *Z*-scores prior to computing odds ratios.

Figure 2 shows an AOR forest plot containing the top 15 features, ranked in descending order by absolute log-odds coefficient. The top five variables included nursing assessments for respiratory effort, tissue perfusion and oxygenation, and neurological WDL check, and the VA medication class for bronchodilators. eFigures 3, 4, and 5 illustrate variable importance for the component horizon models of the F-WIN ensemble by showing the top 15 features for each model, based on their mean absolute SHAP values across the external validation dataset predictions. For the 24-hour horizon model, the five most important variables were the vital signs for Sp02 and respiratory rate and the nursing assessments for tissue perfusion and oxygenation, respiratory effort, and Braden QD score.

In both analyses, the top predictive variables aligned with common pathways of pediatric deterioration, indicating early and subtle signs neurologic changes, respiratory insufficiency, and sepsis, or shock.

### Model fairness

Performance of full and parsimonious F-WIN models within various demographic groups in the external validation dataset are presented in eTables 8 and 9, respectively. Both the full and parsimonious models have statistically significantly better AUROC performance for those aged 19-24 years versus the reference group aged 2-11 years (*P* < .05), at the 12-hour prediction horizon only. No other AUROC comparisons between demographic groups are statistically significant at a level of *P* < .05, for either model.

### Comparison between F-WIN and currently-used prediction models

**Figure 3.**
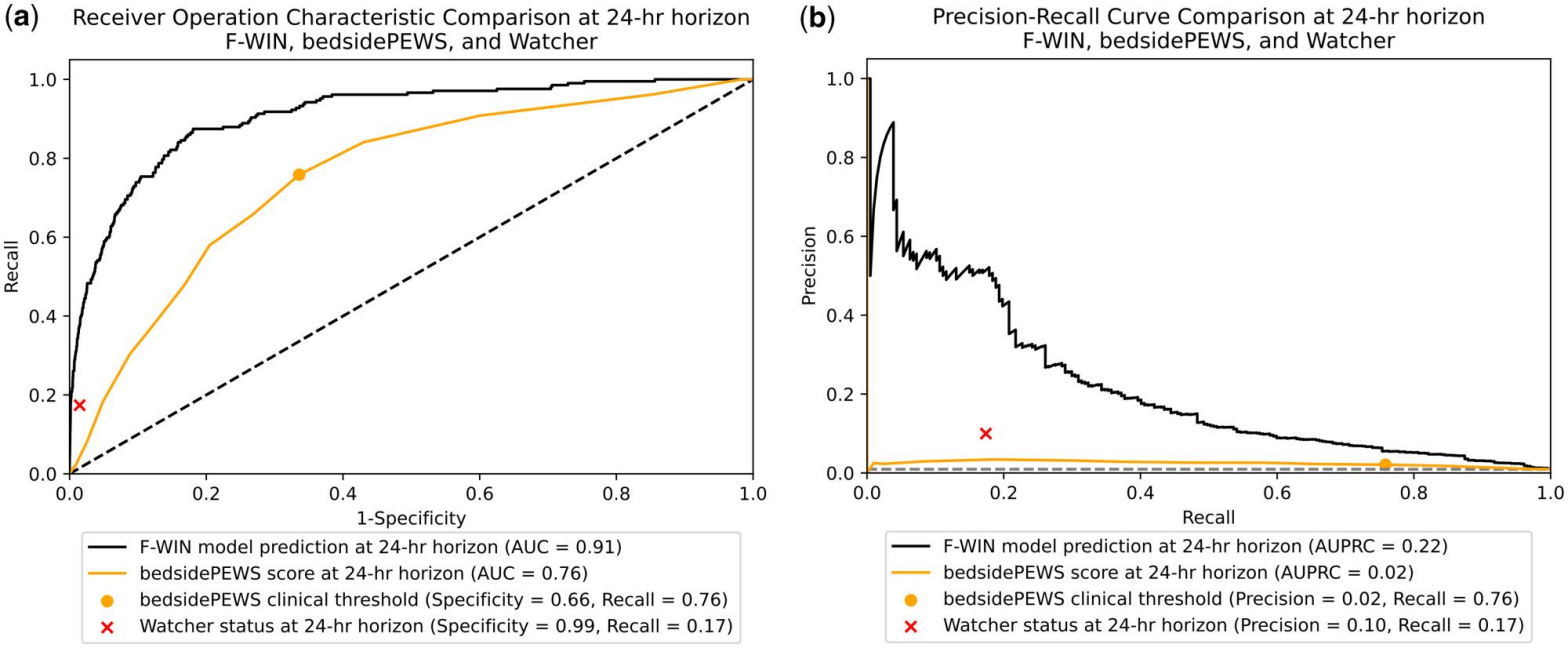
Receiver operating characteristics (ROC) and precision-recall curves (PRC) for the F-WIN model and the bedsidePEWs score, at the 24-hour horizon, in the external validation dataset. We have also marked the performance of the binary Watcher status. The prevalence of unplanned transfer in the external validation dataset was 0.9%.

On the external validation dataset, F-WIN outperformed the bedsidePEWS score, Watcher program, and Clinician Concern score at all prediction horizons. F-WIN achieved higher AUROC and AUPRC than the bedsidePEWS score at all horizons (24-hour AUROC performance: F-WIN 0.91 [95% CI, 0.89-0.93] vs bedsidePEWS 0.76 [95% CI, 0.72-0.79], *P* < .05). Figure 3 shows ROC and PRC curves for F-WIN and bedsidePEWS at the 24-hour horizon, and curves at the 12-hour and 8-hour horizons can be found in eFigure 6. Additionally, F-WIN achieved higher sensitivity, PPV, and F1-score than the Watcher program predictions at comparable specificity, at all horizons on the external validation dataset: (24-hour sensitivity performance: F-WIN 0.31 [95% CI, 0.25-0.38] vs Watcher 0.17 [95% CI, 0.13-0.23], *P* < .05; 24-hour PPV performance: F-WIN 0.23 [95% CI, 0.19-0.28] vs Watcher 0.10 [95% CI, 0.07-0.13], *P* < .05; 24-hour F1-score performance: F-WIN 0.27 [95% CI, 0.22-0.32] vs Watcher 0.13 [95% CI, 0.09-0.16], *P* < .05). Among admissions from 2024-07-01 thru 2024-12-31, F-WIN outperformed the Clinician Concern score (24-hour AUROC performance: F-WIN 0.92 [95% CI, 0.86-0.96] vs Clinician Concern 0.64 [0.58-0.72], *P* < .05). Tables 2, 3 and eTable 10 provide full comparisons between F-WIN and bedsidePEWS, Watcher, and Clinician Concern scores, respectively. F-WIN also attained higher sensitivity, PPV, and F1-score than the binarized bedsidePEWS and Clinician Concern scores, derived using commonly used clinical thresholds, as shown in eTables 11 and 12.

**Table 3.** Comparison between the F-WIN Model and the Watcher Program Scores on the External Validation Dataset.

Prediction horizon	F-WIN Model	Watcher Scores
**24-hour**		
** Sensitivity (95% CI)**	0.31 (0.25-0.38)	0.17 (0.13-0.23)^a^
** Specificity (95% CI)**	0.99 (0.99-0.99)	0.99 (0.98-0.99)
** PPV (95% CI)**	0.23 (0.19-0.28)	0.10 (0.07-0.13)^b^
** F1-score (95% CI)**	0.27 (0.22-0.32)	0.13 (0.09-0.16) ^c^
**12-hour**		
** Sensitivity (95% CI)**	0.39 (0.32-0.46)	0.25 (0.19-0.30)^a^
** Specificity (95% CI)**	0.99 (0.99-0.99)	0.99 (0.98-0.99)
** PPV (95% CI)**	0.27 (0.23-0.31)	0.15 (0.12-0.18) ^b^
** F1-score (95% CI)**	0.32 (0.27-0.37)	0.18 (0.14-0.23) ^c^
**8-hour**		
** Sensitivity (95% CI)**	0.44 (0.37-0.51)	0.29 (0.23-0.35) ^a^
** Specificity (95% CI)**	0.99 (0.99-0.99)	0.99 (0.99-0.99)
** PPV (95% CI)**	0.29 (0.25-0.34)	0.17 (0.14-0.21) ^b^
** F1-score (95% CI)**	0.35 (0.30-0.40)	0.22 (0.17-0.26) ^c^
**2-hour**		
** Sensitivity (95% CI)**	0.60 (0.53-0.66)	0.45 (0.39-0.52)^a^
** Specificity (95% CI)**	0.99 (0.99-0.99)	0.99 (0.99-0.99)
** PPV (95% CI)**	0.36 (0.32-0.40)	0.27 (0.23-0.30) ^b^
** F1-score (95% CI)**	0.45 (0.40-0.50)	0.34 (0.29-0.38) ^c^

The prevalence of ICU transfer with subsequent CDE in the external validation dataset was 0.9%.

The prediction threshold for the F-WIN model was chosen to achieve the same specificity as the Watcher Score predictions at each prediction horizon. The Watcher Score is a binary prediction indicating whether the patient currently had active Watcher status at the prediction horizon. bedsidePEWS is not included here as it has much lower specificity at the clinical threshold.

a Statistically significant difference (McNemar’s test for comparison of sensitivities, *P* < .05) compared with the F-WIN model at the same prediction horizon.

b Statistically significant difference (Kosinski’s weighted generalized test for comparison of PPVs, *P* < .05) compared with the F-WIN model at the same prediction horizon.

c Statistically significant difference (two-sided permutation test for F1-score, *P* < .05) compared with the F-WIN model at the same prediction horizon.

## Discussion

In this single-center study, we developed and validated F-WIN, an ensemble machine learning model that identifies hospitalized children on medical/surgical wards who are likely to experience unplanned transfer to the PICU or NICU followed by critical deterioration within 12 hours, using routinely-collected EHR data. To shift recognition earlier than conventional pediatric early warning systems (PEWS), we targeted prediction horizons up to 24 hours pre-transfer.

F-WIN demonstrated high discrimination and precision in a temporally-distinct external validation cohort and outperformed several baseline alert methods including the bedsidePEWS scores and the Watcher program, at a range of prediction horizons. These findings align with reports that PEWS performance is limited for unplanned ICU transfer,^24^^,^^25^ and show that a data-driven EHR-based ML model can provide better discrimination at longer lead times than PEWS.

We developed a 62-variable parsimonious ensemble model which approximates the performance of F-WIN on the external validation dataset across 8-24 hour prediction horizons. The top variables align with established pathophysiologic pathways of pediatric deterioration, particularly around worsening respiratory status, heightened inflammatory response, neurologic dysfunction, and early signs of circulatory compromise, shock, or sepsis, along with markers of patient fragility.

It is crucial to address the feasibility of deploying the F-WIN model. Medication variables rely on mappings from NDC and Rxnorm codes to Rxnorm active ingredients and VA classes, which would be performed initially prior to implementation. Nursing assessment variables depend on semi-structured data which could vary significantly between institutions. Both of these variable types would need to be derived at a new institution prior to deployment, and updated periodically as the EHR changes.

F-WIN’s tradeoffs in sensitivity and PPV across varied alert thresholds can be balanced to be compatible with various clinical goals and workflows. For example, higher-PPV thresholds may be favored closer to the event to support actionable, high-confidence interventions, whereas higher-sensitivity (lower-PPV) thresholds may be preferred at earlier time points to provide greater lead time for monitoring and clinical decision-making, even at the cost of increased false positives.

Prior work suggests that up to 40% of critical deterioration events may be preventable, underscoring the importance of timely, data-driven interventions, and F-WIN addresses the gap left by other alert methods. The F-WIN model’s AUROC performance at both the 12-hour and 24-hour prediction horizons exceeds the published performance of 0.84 of the pCART model 12 hours in advance, and this was still the case when we expanded the external validation dataset to include floor stays ending in ICU transfer without subsequent CDE. These improvements in timeliness and accuracy offer the potential to reduce post-transfer deterioration, improve patient safety, and lower downstream mortality.

## Limitations

This study has several limitations. F-WIN was developed based on a single-center cohort, which allowed for strong internal validity but may limit its generalizability. Evaluating our model on data from an external site will be essential to confirming its ability to predict unplanned ICU transfer in diverse settings, and perhaps the most effective path for implementation is a federated system incorporating strong locally-attuned models.^26^ Second, our performance evaluation and model fairness testing were entirely retrospective. A key next step would be to perform prospective evaluation including ongoing monitoring of performance and demographic bias. Third, our cohort did not contain patients who were transferred to the CCU or ITCU, or patients who had an active DNR order in place, so performance is unknown in these settings. Fourth, due to our limitation to floor stays at least 30 hours in length (resulting in an exclusion of approximately 45% of patients), the model’s predictive performance for patients with shorter hospital stays is not known. Finally, as with any data-driven ML model, F-WIN is vulnerable to model drift due to changes in variable distributions and patient mix. Ongoing deployment may necessitate that the model be recalibrated or updated over time.

## Future work

We are currently conducting a silent-mode prospective deployment of the model to evaluate its robustness and prospective predictive performance. We plan to develop the clinician-facing interface and embed the system within the EHR system to fit clinicians’ workflow. The necessary data elements for the F-WIN model for all patients admitted to floor departments will be extracted in real time via a combination of Fast Healthcare Interoperability Resources (FHIR)^27^ and proprietary EPIC API pipelines.^28^

## Conclusion

An ML model developed to predict floor-to-ICU transfer followed by clinical deterioration, F-WIN, delivers high discrimination up to 24 hours in advance of the transfer. F-WIN outperforms several existing alert methods, including bedsidePEWS scores. Future work will include evaluation at an external institution as well as prospective evaluation and ongoing monitoring of performance and model fairness.

## Supplementary Material

ooag105_Supplementary_Data

## Data Availability

The data used in this study are owned by CHOP and contain sensitive patient information. Due to institutional and privacy regulations, these data cannot be shared publicly. Researchers interested in accessing the data must obtain approval from CHOP and comply with all applicable data use agreements and ethical guidelines. The data dictionary can be shared upon request. The underlying code for this study is not publicly available but may be made available to qualified researchers on reasonable request from the corresponding author, pending CHOP approval.
