# Supplementary material for: Early prediction of unplanned critical care transfers in children using EHR-based ensemble machine learning
Source: JAMIA Open. 2026 Jun 13;9(3):ooag105. doi: 10.1093/jamiaopen/ooag105 (PMC13264393; doi:10.1093/jamiaopen/ooag105)
Supplement: ooag105_Supplementary_Data [file ooag105_Supplementary_Data.docx]

Supplemental Digital Content

Table of Contents

[eMethods 2](#_Toc227911438)

[Details about feature engineering 2](#_Toc227911439)

[eFigures 3](#_Toc227911440)

[**eFigure 1**. Plots of binary metrics for the F-WIN model over a range of prediction thresholds, on the external validation dataset, at the 24-hour (a), 12-hour (b), and 8-hour (c) horizons. The prevalence of events in the external validation dataset was 0.9%. 3](#_Toc227911441)

[**eFigure 2**. Receiver operating characteristics (ROC) and precision-recall curves (PRC) for the F-WIN model (a,b) and the 62-variable Parsimonious XGB ensemble model (c,d), on the external validation dataset. The prevalence of events in the external validation dataset was 0.9%. 4](#_Toc227911442)

[**eFigure 3**. SHapley Additive exPlanations (SHAP) value results for the predictions of the XGBoost F-WIN 24-hour horizon model on the external validation dataset. We include the top 15 features, ranked by mean absolute SHAP value across this dataset. 5](#_Toc227911443)

[**eFigure 4**. SHapley Additive exPlanations (SHAP) value results for the predictions of the XGBoost F-WIN 12-hour horizon model on the external validation dataset. We include the top 15 features, ranked by mean absolute SHAP value across this dataset. 6](#_Toc227911444)

[**eFigure 5**. SHapley Additive exPlanations (SHAP) value results for the predictions of the XGBoost F-WIN 8-hour horizon model on the external validation dataset. We include the top 15 features, ranked by mean absolute SHAP value across this dataset. 7](#_Toc227911445)

[**eFigure 6.** Receiver operating characteristics (ROC) and precision-recall curves (PRC) for the F-WIN model and the bedsidePEWs score, at the 12-hour horizon (a,b) and the 8-hour horizon (c,d), in the external validation dataset. We have also marked the performance of the binary Watcher status. The prevalence of unplanned transfer in the external validation dataset was 0.9%. 8](#_Toc227911446)

[eTables 8](#_Toc227911447)

[**eTable 1.** Counts of Critical Deterioration Event (CDE) types among the development and external validation datasets. 8](#_Toc227911448)

[**eTable 2.** Patient-level demographic characteristics and odds ratios for the F-WIN development dataset. 9](#_Toc227911449)

[**eTable 3.** Patient-level demographic characteristics and odds ratios for the F-WIN external validation dataset. 9](#_Toc227911450)

[**eTable 4.** Cross-validation performance of the XGBoost, Random Forest, and LASSO ensemble models on the development dataset. 10](#_Toc227911451)

[**eTable 5.** Cross-validation performance of parsimonious XGBoost models on the development dataset. 10](#_Toc227911452)

[**eTable 6**.Sensitivity, specificity, and positive predictive values (PPV) for the F-WIN model on the external validation dataset, at six prediction thresholds. 11](#_Toc227911453)

[**eTable 7.** F-WIN performance on the expanded external validation dataset, which included stays with ICU transfer not followed by CDE. 11](#_Toc227911454)

[**eTable 8.** Model fairness assessment for the F-WIN model on the external validation dataset. 12](#_Toc227911455)

[**eTable 9.** Model fairness assessment for the 62-feature F-WIN parsimonious model on the external validation dataset. 12](#_Toc227911456)

[**eTable 10.** Comparison between the F-WIN Model and the Clinician Concern Score on admissions between 2024-07-1 and 2024-12-31. 13](#_Toc227911457)

[**eTable 11.** Comparison between the F-WIN Model and binarized Bedside PEWS predictions on the External Validation Dataset. 13](#_Toc227911458)

[**eTable 12.** Comparison between the F-WIN Model and binarized Clinician Concern prediction on admissions between 2024-07-1 and 2024-12-31. 14](#_Toc227911459)

[TRIPOD+AI Checklist 15](#_Toc227911460)

[STROBE Checklist 17](#_Toc227911461)

[References 19](#_Toc227911462)

## eMethods

### Details about feature engineering

Demographic variables consisted of age, sex, race, and ethnicity. Diagnosis variables consisted of binary indicators based on International Classification of Diseases, 10^th^ Edition (ICD-10) codes seen in the patient’s 4-year history prior to the present hospital encounter. To limit the feature space, we only used the portion of each ICD-10 code appearing before the decimal. Medications were mapped from National Drug Codes (NDC) to RxNorm^1^ active ingredients and Veterans Administration (VA) drug classes^2^ using the RxNav tool hosted by the National Library of Medicine at the National Institutes of Health (<https://lhncbc.nlm.nih.gov/RxNav/>). The resulting active ingredient and VA class variables were binary, indicating whether corresponding medication(s) were administered during the 4 hours prior to the prediction time.

For each laboratory test and numerical vital sign element, we aggregated the time series of results into hourly averaged values and subsequently defined 14 variables from each aggregated time series: (1) the first value, (2) the last value, (3) the maximum value, (4) the minimum value, (5) the difference between the last two values, (6) the difference between the last two values divided by the last value, (7) the difference between the last value and the maximum value, (8) the difference between the last value and the minimum value, (9) the difference between the last value and the maximum value, divided by the maximum value, (10) the difference between the last value and the minimum value, divided by the minimum value, (11) the difference between the first value and the last value, (12) the difference between the first value and the last value, divided by the first value, (13) the slope of the last two values, and (14) the linear regression slope from all values.

Nursing assessment data were semi-structured, as some elements have a result that is a semicolon-separated list of multiple entries. To process such nursing assessment elements, we split the lists and stacked the split values into multiple records with the same timestamp. Then we selected the most recent record(s) for each element prior to the prediction time and represented them via dummy encoding.

## eFigures

**eFigure 1**. Plots of binary metrics for the F-WIN model over a range of prediction thresholds, on the external validation dataset, at the 24-hour (a), 12-hour (b), and 8-hour (c) horizons. The prevalence of events in the external validation dataset was 0.9%.

**eFigure 2**. Receiver operating characteristics (ROC) and precision-recall curves (PRC) for the F-WIN model (a,b) and the 62-variable Parsimonious XGB ensemble model (c,d), on the external validation dataset. The prevalence of events in the external validation dataset was 0.9%.

***
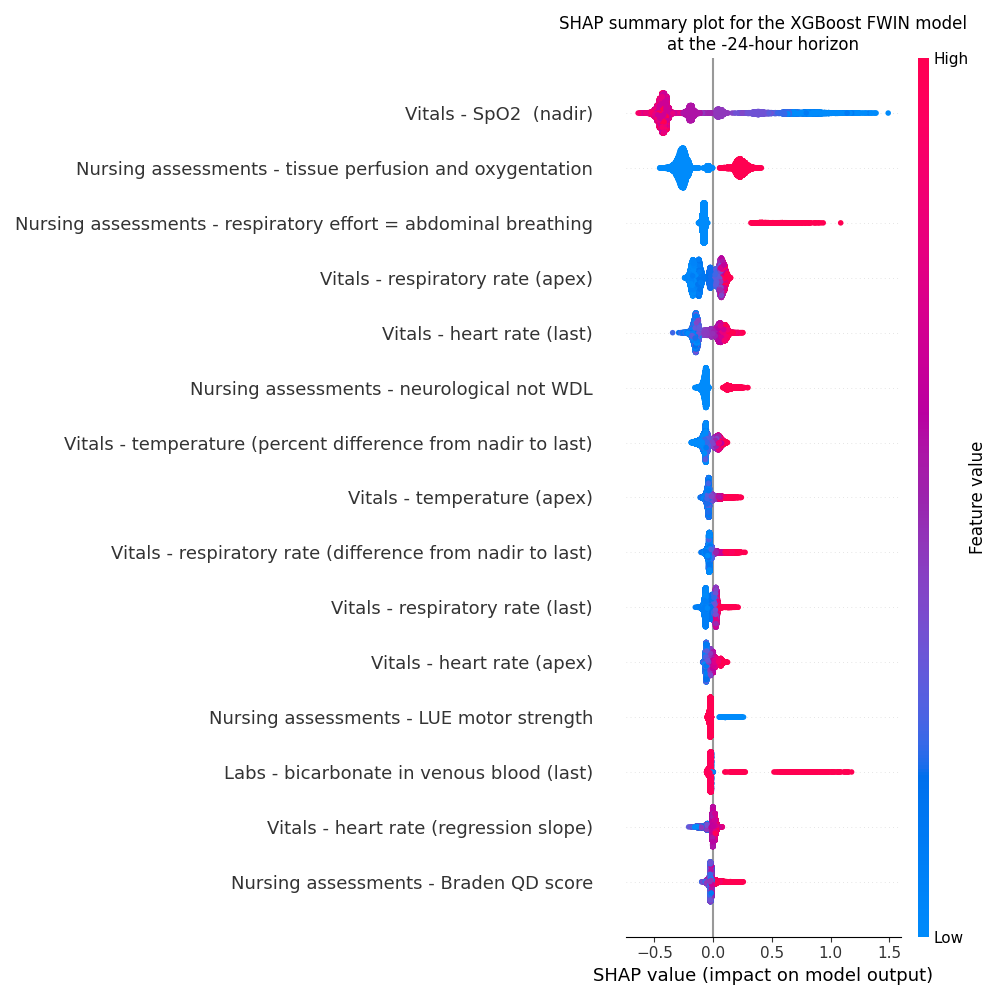
***

**eFigure 3**. SHapley Additive exPlanations (SHAP) value results for the predictions of the XGBoost F-WIN 24-hour horizon model on the external validation dataset. We include the top 15 features, ranked by mean absolute SHAP value across this dataset.

*
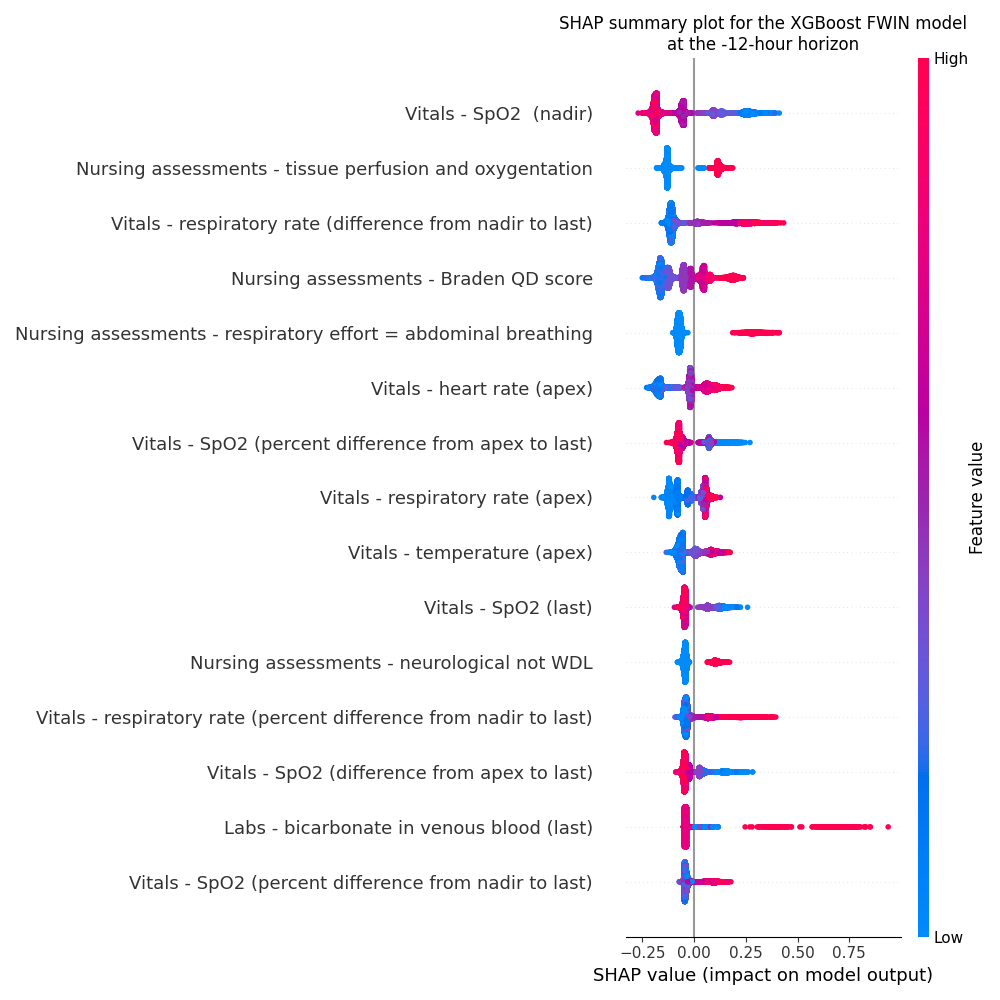
*

**eFigure 4**. SHapley Additive exPlanations (SHAP) value results for the predictions of the XGBoost F-WIN 12-hour horizon model on the external validation dataset. We include the top 15 features, ranked by mean absolute SHAP value across this dataset.

*
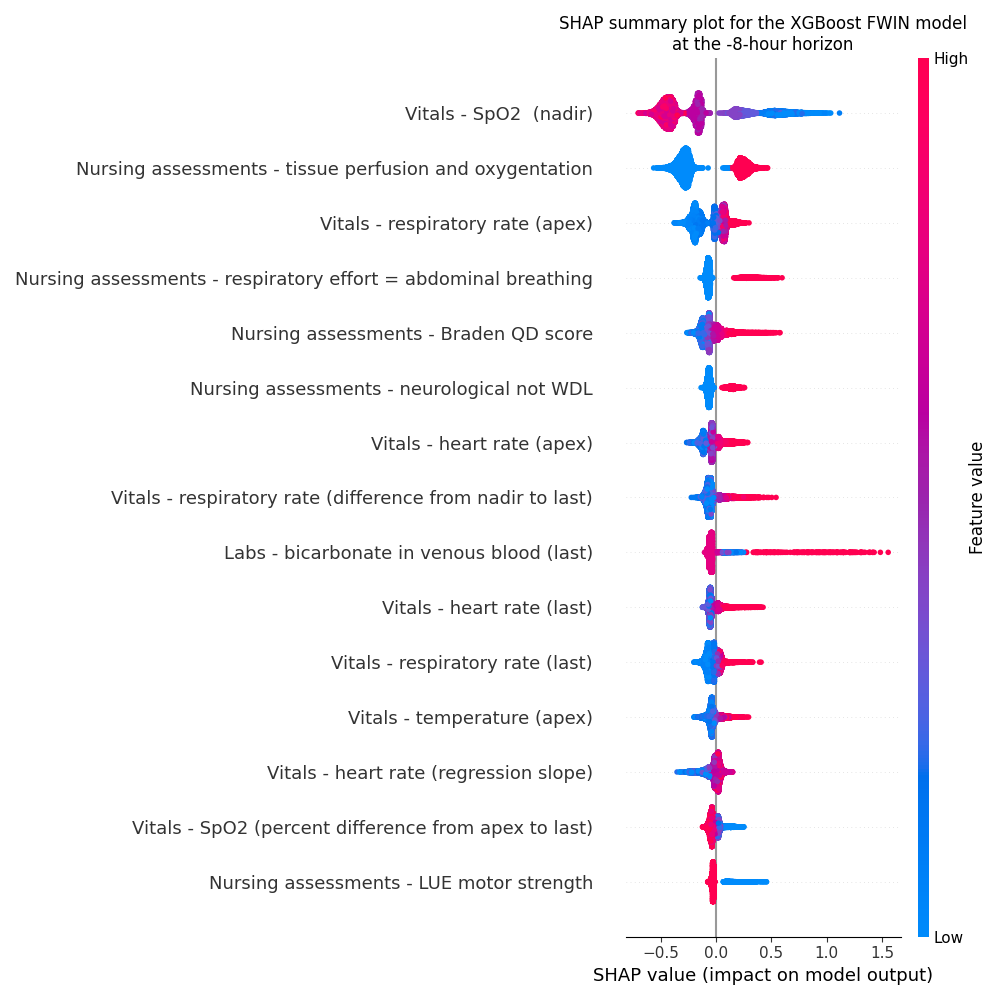
*

**eFigure 5**. SHapley Additive exPlanations (SHAP) value results for the predictions of the XGBoost F-WIN 8-hour horizon model on the external validation dataset. We include the top 15 features, ranked by mean absolute SHAP value across this dataset.

******

**eFigure 6.** Receiver operating characteristics (ROC) and precision-recall curves (PRC) for the F-WIN model and the bedsidePEWs score, at the 12-hour horizon (a,b) and the 8-hour horizon (c,d), in the external validation dataset. We have also marked the performance of the binary Watcher status. The prevalence of unplanned transfer in the external validation dataset was 0.9%.

## eTables

**eTable 1.** Counts of Critical Deterioration Event (CDE) types among the development and external validation datasets.

|  | Type of earliest CDE following transfer to the ICU | | | |
| --- | --- | --- | --- | --- |
|  | Intubation | New non-invasive ventilation | Administration of vasopressors or inotropes | Death |
| Development dataset  (2019-2022) | 332 | 221 | 55 | 1 |
| External validation dataset (2023-2024) | 84 | 105 | 17 | 1 |

Breakdown of the type of the earliest CDE experienced within 12 hours following an ICU transfer for stays in the development dataset and external validation dataset, respectively.

**eTable 2.** Patient-level demographic characteristics and odds ratios for the F-WIN development dataset.

|  | Counts of patients who did and did not experience a transfer from the floor to the ICU followed by CDE^c^ | | |
| --- | --- | --- | --- |
|  | **Did not experience ICU transfer with CDE (%)**  **[n = 31938]** | **Experienced ICU transfer with CDE (%)**  **[n = 348]** | **Odds Ratio**  **(95% CI)** |
| **Sex** |  |  |  |
| Female | 15838 (49.6) | 172 (49.4) | 0.99 (0.8-1.23) |
| Male | 16097 (50.4) | 176 (50.6) | 1 [Reference] |
| Unknown | 3 (0.0) | 0 (0.0) | NA |
| **Race** |  |  |  |
| Black | 8641 (27.1) | 94 (27.0) | 1.14 (0.88-1.47) |
| White | 16407 (51.4) | 157 (45.1) | 1 [Reference] |
| Other^a^ | 6089 (19.1) | 88 (25.3) | 1.51 (1.16-1.96) |
| Unknown^b^ | 801 (2.5) | 9 (2.6) | 1.17 (0.55-2.17) |
| **Hispanic or Latino Ethnicity** |  |  |  |
| Yes | 4150 (13.0) | 74 (21.3) | 1.82 (1.39-2.34) |
| No | 27418 (85.8) | 269 (77.3) | 1 [Reference] |
| Unknown^b^ | 370 (1.2) | 5 (1.4) | 1.38 (0.49-3.01) |
| **Age** |  |  |  |
| < 28 d | 2280 (7.1) | 47 (13.5) | 2.16 (1.52-3.04) |
| At least 28 d, < 2 yr | 7327 (22.9) | 132 (37.9) | 1.89 (1.46-2.45) |
| 2-11 yr | 10919 (34.2) | 104 (29.9) | 1 [Reference] |
| 12-18 yr | 10490 (32.8) | 56 (16.1) | 0.56 (0.4-0.77) |
| 19-24 yr | 922 (2.9) | 9 (2.6) | 1.02 (0.48-1.92) |

Demographic fields from the EHR were collected during the first hospitalization for each patient.

^a^The “Other” race category contains patients whose self-reported race in the EHR was “Asian”, “Indian”, “Native Hawaiian or Other Pacific Islander”, “American Indian or Alaska Native”, or “Other”.

^b^The “Unknown” Race and Ethnicity categories comprise patients whose self-reported race in the EHR was “Choose note to disclose”, “Unknown”, “Asked but unknown”, or “Refused”, or who had multiple conflicting responses during the hospitalization.

^c^Patient-level outcomes are based on whether that patient experienced an ICU transfer followed by CDE during their first floor stay in the dataset.

**eTable 3.** Patient-level demographic characteristics and odds ratios for the F-WIN external validation dataset.

|  | **Counts of patients who did and did not experience a transfer from the floor to the ICU followed by CDE^c^** | | |
| --- | --- | --- | --- |
|  | **Did not experience ICU transfer with CDE (%)**  **[n = 16858]** | **Experienced ICU transfer with CDE (%)**  **[n = 139]** | **Odds Ratio**  **(95% CI)** |
| **Sex** |  |  |  |
| Female | 8200 (48.6) | 56 (40.3) | 0.71 (0.5-1) |
| Male | 8657 (51.4) | 83 (59.7) | 1 [Reference] |
| Unknown | 1 (0.0) | 0 (0.0) | NA |
| **Race** |  |  |  |
| Black | 3862 (22.9) | 34 (24.5) | 1.19 (0.78-1.8) |
| White | 8677 (51.5) | 64 (46.0) | 1 [Reference] |
| Other^a^ | 3615 (21.4) | 34 (24.5) | 1.28 (0.83-1.92) |
| Unknown^b^ | 704 (4.2) | 7 (5.0) | 1.35 (0.56-2.75) |
| **Hispanic or Latino Ethnicity** |  |  |  |
| Yes | 2575 (15.3) | 25 (18.0) | 1.27 (0.8-1.93) |
| No | 13874 (82.3) | 106 (76.3) | 1 [Reference] |
| Unknown^b^ | 409 (2.4) | 8 (5.8) | 2.56 (1.14-4.96) |
| **Age** |  |  |  |
| < 28 d | 1121 (6.6) | 20 (14.4) | 2.91 (1.65-4.97) |
| At least 28 d, < 2 yr | 4126 (24.5) | 52 (37.4) | 2.05 (1.35-3.16) |
| 2-11 yr | 6027 (35.8) | 37 (26.6) | 1 [Reference] |
| 12-18 yr | 5256 (31.2) | 29 (20.9) | 0.9 (0.55-1.46) |
| 19-24 yr | 328 (1.9) | 1 (0.7) | 0.5 (0.03-2.3) |

Demographic fields from the EHR were collected during the first hospitalization for each patient.

^a^The “Other” race category contains patients whose self-reported race in the EHR was “Asian”, “Indian”, “Native Hawaiian or Other Pacific Islander”, “American Indian or Alaska Native”, or “Other”.

^b^The “Unknown” Race and Ethnicity categories comprise patients whose self-reported race in the EHR was “Choose note to disclose”, “Unknown”, “Asked but unknown”, or “Refused”, or who had multiple conflicting responses during the hospitalization.

^c^Patient-level outcomes are based on whether that patient experienced an ICU transfer followed by CDE during their first floor stay in the dataset.

**eTable 4.** Cross-validation performance of the XGBoost, Random Forest, and LASSO ensemble models on the development dataset.

| **Prediction horizon** | **XGB model** | **RF model** | **LASSO model** |
| --- | --- | --- | --- |
| **24-hour** |  |  |  |
| AUROC (95% CI) | 0.90 (0.89-0.91)^b^ | 0.89 (0.88-0.91)^a^ | 0.89 (0.88-0.91) ^a^ |
| AUPRC (95% CI) | 0.22 (0.19-0.26) | 0.20 (0.17-0.23) | 0.19 (0.16-0.22) |
| **12-hour** |  |  |  |
| AUROC (95% CI) | 0.93 (0.92-0.94) ^b^ | 0.92 (0.91-0.93) ^a^ | 0.91 (0.90-0.93) ^a^ |
| AUPRC (95% CI) | 0.32 (0.28-0.36) | 0.28 (0.24-0.31) | 0.25 (0.22-0.28) |
| **8-hour** |  |  |  |
| AUROC (95% CI) | 0.93 (0.92-0.94) ^b^ | 0.92 (0.91-0.93) ^a^ | 0.92 (0.91-0.93) ^a^ |
| AUPRC (95% CI) | 0.35 (0.31-0.39) | 0.30 (0.27-0.34) | 0.27 (0.24-0.31) |

The prevalence of ICU transfer with subsequent CDE in the development dataset was 1.2%.

^a^Statistically significant difference (Delong test P<0.05) compared with the XGB model at the same prediction horizon.

^b^Indicates the best performance across models within a fixed prediction horizon.

**eTable 5.** Cross-validation performance of parsimonious XGBoost models on the development dataset.

| **Prediction horizon** | **k = 20 model** | **k = 40 model** | **k = 60 model** |
| --- | --- | --- | --- |
| **24-hour** |  |  |  |
| AUROC (95% CI) | 0.84 (0.82-0.86) ^a^ | 0.87 (0.85-0.88) ^a^ | 0.89 (0.87-0.90) ^b^ |
| AUPRC (95% CI) | 0.14 (0.12-0.17) | 0.18 (0.15-0.21) | 0.21 (0.18-0.25) |
| **12-hour** |  |  |  |
| AUROC (95% CI) | 0.87 (0.86-0.89) ^a^ | 0.90 (0.88-0.91) ^a^ | 0.91 (0.90-0.92) ^b^ |
| AUPRC (95% CI) | 0.19 (0.17-0.23) | 0.25 (0.22-0.28) | 0.29 (0.26-0.32) |
| **8-hour** |  |  |  |
| AUROC (95% CI) | 0.88 (0.86-0.89) ^a^ | 0.90 (0.89-0.92) ^a^ | 0.92 (0.90-0.93) ^b^ |
| AUPRC (95% CI) | 0.22 (0.19-0.25) | 0.28 (0.25-0.32) | 0.33 (0.29-0.37) |

Model pipelines were fit using a top-k univariate feature selection step based on mutual information with the outcome, for k-values of 20, 40, and 60, respectively.

The prevalence of ICU transfer with subsequent CDE in the development dataset was 1.2%.

^a^Statistically significant difference (Delong test P<0.05) compared with the k = 60 model at the same prediction horizon,

^b^Indicates the best performance across models within a fixed prediction horizon.

**eTable 6**.Sensitivity, specificity, and positive predictive values (PPV) for the F-WIN model on the external validation dataset, at six prediction thresholds.

| **Targeting sensitivity = 0.90** | | | |  |
| --- | --- | --- | --- | --- |
| Prediction horizon | Sensitivity (95% CI) | Specificity (95% CI) | PPV (95% CI) | F1-score (95% CI) |
| 24-hour | 0.90 (0.86 to 0.94) | 0.73 (0.73 to 0.74) | 0.03 (0.03 to 0.03) | 0.06 (0.06 to 0.06) |
| 12-hour | 0.93 (0.90 to 0.97) | 0.71 (0.70 to 0.71) | 0.03 (0.03 to 0.03) | 0.06 (0.05 to 0.06) |
| 8-hour | 0.93 (0.90 to 0.97) | 0.71 (0.70 to 0.71) | 0.03 (0.03 to 0.03) | 0.06 (0.05 to 0.06) |
| **Targeting sensitivity = 0.80** | | | |  |
| Prediction horizon | Sensitivity (95% CI) | Specificity (95% CI) | PPV (95% CI) | F1-score (95% CI) |
| 24-hour | 0.81 (0.75 to 0.86) | 0.86 (0.86 to 0.87) | 0.05 (0.05 to 0.06) | 0.10 (0.09 to 0.10) |
| 12-hour | 0.86 (0.81 to 0.91) | 0.85 (0.84 to 0.85) | 0.05 (0.05 to 0.05) | 0.09 (0.09 to 0.10) |
| 8-hour | 0.88 (0.84 to 0.93) | 0.85 (0.85 to 0.86) | 0.05 (0.05 to 0.06) | 0.10 (0.09 to 0.11) |
| **Targeting sensitivity = 0.50** | | | | |
| Prediction horizon | Sensitivity (95% CI) | Specificity (95% CI) | PPV (95% CI) | F1-score (95% CI) |
| 24-hour | 0.50 (0.43 to 0.57) | 0.97 (0.96 to 0.97) | 0.12 (0.11 to 0.14) | 0.20 (0.17 to 0.22) |
| 12-hour | 0.65 (0.58 to 0.71) | 0.97 (0.96 to 0.97) | 0.16 (0.14 to 0.17) | 0.25 (0.22 to 0.28) |
| 8-hour | 0.68 (0.61 to 0.74) | 0.97 (0.97 to 0.97) | 0.17 (0.16 to 0.19) | 0.27 (0.25 to 0.30) |
| **Targeting specificity = 0.80** | | | |  |
| Prediction horizon | Sensitivity (95% CI) | Specificity (95% CI) | PPV (95% CI) | F1-score (95% CI) |
| 24-hour | 0.87 (0.83 to 0.92) | 0.80 (0.80 to 0.81) | 0.04 (0.04 to 0.04) | 0.08 (0.07 to 0.08) |
| 12-hour | 0.92 (0.88 to 0.95) | 0.78 (0.77 to 0.79) | 0.04 (0.04 to 0.04) | 0.07 (0.07 to 0.08) |
| 8-hour | 0.92 (0.88 to 0.95) | 0.78 (0.78 to 0.79) | 0.04 (0.04 to 0.04) | 0.07 (0.07 to 0.08) |
| **Targeting specificity = 0.90** | | | |  |
| Prediction horizon | Sensitivity (95% CI) | Specificity (95% CI) | PPV (95% CI) | F1-score (95% CI) |
| 24-hour | 0.74 (0.68 to 0.80) | 0.90 (0.90 to 0.90) | 0.07 (0.06 to 0.07) | 0.12 (0.11 to 0.13) |
| 12-hour | 0.81 (0.75 to 0.86) | 0.89 (0.89 to 0.90) | 0.07 (0.06 to 0.07) | 0.12 (0.11 to 0.13) |
| 8-hour | 0.86 (0.81 to 0.91) | 0.90 (0.89 to 0.90) | 0.07 (0.07 to 0.08) | 0.14 (0.13 to 0.14) |
| **Targeting specificity = 0.95** | | | |  |
| Prediction horizon | Sensitivity (95% CI) | Specificity (95% CI) | PPV (95% CI) | F1-score (95% CI) |
| 24-hour | 0.58 (0.51 to 0.65) | 0.95 (0.95 to 0.95) | 0.10 (0.09 to 0.11) | 0.17 (0.15 to 0.19) |
| 12-hour | 0.70 (0.63 to 0.76) | 0.95 (0.95 to 0.95) | 0.12 (0.11 to 0.13) | 0.20 (0.18 to 0.22) |
| 8-hour | 0.74 (0.68 to 0.80) | 0.95 (0.95 to 0.96) | 0.13 (0.12 to 0.14) | 0.22 (0.20 to 0.24) |

The prevalence of ICU transfer with subsequent CDE in the external validation dataset was 0.9%.

The six F-WIN prediction thresholds were chosen to achieve sensitivity or 0.50, 0.80, or 0.90, or specificity of 0.8, 0.9, or 0.95, respectively, at the 24-hour horizon.

**eTable 7.** F-WIN performance on the expanded external validation dataset, which included stays with ICU transfer not followed by CDE.

|  | **24-Hour Horizon** | | **12-Hour Horizon** | | **8-Hour Horizon** | |
| --- | --- | --- | --- | --- | --- | --- |
|  | **AUROC**  **(95% CI)** | **AUPRC**  **(95% CI)** | **AUROC**  **(95% CI)** | **AUPRC**  **(95% CI)** | **AUROC**  **(95% CI)** | **AUPRC**  **(95% CI)** |
| External validation dataset (reference) | 0.91  (0.89-0.93) | 0.22  (0.17-0.29) | 0.92  (0.90-0.94) | 0.28  (0.22-0.35) | 0.93  (0.91-0.95) | 0.36  (0.29-0.43) |
| Expanded external validation dataset | 0.91  (0.88-0.92) | 0.18  (0.14-0.23) | 0.92  (0.90-0.94) | 0.22  (0.17-0.28) | 0.93  (0.91-0.95) | 0.28  (0.22-0.34) |

The expanded external validation dataset included 241 additional stays in 2023-2024 which end in transfer to the ICU without any subsequent CDE.

The prevalence of ICU transfer followed by CDE in both the external validation and expanded external validation datasets was 0.9%.

At each of the three prediction horizons, the AUROC performance was not statistically significantly different at a level of P < 0.05 (Delong test).

**eTable 8.** Model fairness assessment for the F-WIN model on the external validation dataset.

| **Variable** | **AUROC (95% CI) at 24h** | **AUROC (95% CI) at 12h** | **AUROC (95% CI) at 8h** |
| --- | --- | --- | --- |
| **Sex** |  |  |  |
| Female | 0.93 (0.90 - 0.95) | 0.95 (0.93 - 0.97) | 0.96 (0.93 - 0.98) |
| Male^r^ | 0.89 (0.86 - 0.92) | 0.90 (0.86 - 0.93) | 0.92 (0.89 - 0.94) |
| **Race** |  |  |  |
| Black | 0.94 (0.91 - 0.96) | 0.96 (0.93 - 0.97) | 0.96 (0.94 - 0.98) |
| White^r^ | 0.92 (0.89 - 0.95) | 0.93 (0.90 - 0.96) | 0.94 (0.91 - 0.97) |
| Other | 0.87 (0.82 - 0.91) | 0.89 (0.83 - 0.93) | 0.91 (0.86 - 0.95) |
| Unknown | 0.79 (0.62 - 0.93) | 0.88 (0.70 - 0.98) | 0.88 (0.72 - 0.98) |
| **Hispanic or Latino Ethnicity** |  |  |  |
| Yes | 0.88 (0.82 - 0.93) | 0.90 (0.83 - 0.95) | 0.92 (0.86 - 0.97) |
| No^r^ | 0.92 (0.89 - 0.94) | 0.93 (0.90 - 0.95) | 0.94 (0.92 - 0.96) |
| Unknown | 0.89 (0.77 - 0.97) | 0.96 (0.91 - 0.99) | 0.95 (0.89 - 1.00) |
| **Age** |  |  |  |
| < 28 days | 0.90 (0.83 - 0.95) | 0.89 (0.79 - 0.97) | 0.89 (0.79 - 0.97) |
| At least 28 days, < 2 years | 0.88 (0.84 - 0.91) | 0.91 (0.89 - 0.94) | 0.94 (0.91 - 0.96) |
| 2-11 years^r^ | 0.90 (0.85 - 0.95) | 0.91 (0.86 - 0.96) | 0.93 (0.88 - 0.97) |
| 12-18 years | 0.93 (0.89 - 0.96) | 0.93 (0.88 - 0.97) | 0.95 (0.91 - 0.98) |
| 19-24 years | 0.96 (0.88 - 1.00) | 1.00 (0.98 - 1.00) ^a^ | 0.99 (0.97 - 1.00) |

The full F-WIN XGB ensemble model’s AUROC scores when restricted to individual demographic groups of the external validation dataset, at each prediction horizon. Groups containing fewer than 5 samples are omitted.

^r^Reference group for each demographic variable.

^a^Statistically significant difference (Delong test P<0.05) compared with the reference group of the corresponding demographic variable at the same prediction horizon.

**eTable 9.** Model fairness assessment for the 62-feature F-WIN parsimonious model on the external validation dataset.

| **Variable** | **AUROC (95% CI) at 24h** | **AUROC (95% CI) at 12h** | **AUROC (95% CI) at 8h** |
| --- | --- | --- | --- |
| **Sex** |  |  |  |
| Female | 0.92 (0.89 - 0.95) | 0.94 (0.91 - 0.96) | 0.95 (0.92 - 0.97) |
| Male^r^ | 0.89 (0.86 - 0.91) | 0.89 (0.86 - 0.93) | 0.91 (0.88 - 0.94) |
| **Race** |  |  |  |
| Black | 0.93 (0.91 - 0.95) | 0.95 (0.92 - 0.97) | 0.95 (0.93 - 0.98) |
| White^r^ | 0.92 (0.89 - 0.94) | 0.92 (0.89 - 0.95) | 0.94 (0.91 - 0.96) |
| Other | 0.87 (0.82 - 0.91) | 0.89 (0.83 - 0.93) | 0.91 (0.86 - 0.94) |
| Unknown | 0.74 (0.51 - 0.92) | 0.81 (0.56 - 0.97) | 0.82 (0.57 - 0.97) |
| **Hispanic or Latino Ethnicity** |  |  |  |
| Yes | 0.87 (0.83 - 0.90) | 0.89 (0.83 - 0.95) | 0.92 (0.86 - 0.96) |
| No^r^ | 0.91 (0.89 - 0.93) | 0.92 (0.89 - 0.94) | 0.93 (0.90 - 0.95) |
| Unknown | 0.90 (0.80 - 0.98) | 0.97 (0.93 - 0.99) | 0.97 (0.93 - 1.00) |
| **Age** |  |  |  |
| < 28 days | 0.89 (0.83 - 0.94) | 0.89 (0.82 - 0.95) | 0.89 (0.81 - 0.95) |
| >= 28 days, < 2 years | 0.87 (0.83 - 0.90) | 0.90 (0.87 - 0.93) | 0.92 (0.89 - 0.94) |
| 2-11 years^r^ | 0.90 (0.86 - 0.94) | 0.91 (0.86 - 0.95) | 0.93 (0.88 - 0.96) |
| 12-18 years | 0.92 (0.87 - 0.96) | 0.91 (0.84 - 0.97) | 0.93 (0.87 - 0.97) |
| 19-24 years | 0.97 (0.92 - 1.00) | 0.99 (0.97 - 1.00) ^a^ | 0.99 (0.97 - 1.00) |

The 62-feature parsimonious F-WIN XGB ensemble model’s AUROC scores when restricted to individual demographic groups of the external validation dataset, at each prediction horizon. Groups containing fewer than 5 samples are omitted.

^r^Reference group for each demographic variable.

^a^Statistically significant difference (Delong test P<0.05) compared with the reference group of the corresponding demographic variable at the same prediction horizon.

**eTable 10.** Comparison between the F-WIN Model and the Clinician Concern Score on admissions between 2024-07-1 and 2024-12-31.

| **Prediction horizon** | **F-WIN Model** | **Clinician Concern Score** |
| --- | --- | --- |
| **24-hour** |  |  |
| AUROC (95% CI) | 0.92 (0.86-0.96) | 0.64 (0.58-0.72) ^a^ |
| AUPRC (95% CI) | 0.22 (0.12-0.36) | 0.02 (0.01-0.04) |
| **12-hour** |  |  |
| AUROC (95% CI) | 0.93 (0.87-0.97) | 0.66 (0.58-0.73) ^a^ |
| AUPRC (95% CI) | 0.30 (0.18-0.45) | 0.02 (0.01-0.04) |
| **8-hour** |  |  |
| AUROC (95% CI) | 0.93 (0.88-0.97) | 0.65 (0.57-0.73) ^a^ |
| AUPRC (95% CI) | 0.34 (0.22-0.50) | 0.03 (0.01-0.05) |

The prevalence of ICU transfer with subsequent CDE for admissions between 2024-07-01 and 2024-12-31 was 0.9%.

The Clinician Concern Score consisted of the maximum Clinician Concern level (0 = No Concern, 1 = Slight Concern, 2 = Moderate Concern, 3 = Significant Concern) seen in the 24-hour period prior to each prediction horizon.

^a^Statistically significant difference (Delong test P<0.05) compared with the F-WIN model at the same prediction horizon.

**eTable 11.** Comparison between the F-WIN Model and binarized Bedside PEWS predictions on the External Validation Dataset.

| **Prediction horizon** | **F-WIN Model** | **Binarized Bedside PEWS** |
| --- | --- | --- |
| **24-hour** |  |  |
| Sensitivity (95% CI) | 0.93 (0.90-0.97) | 0.76 (0.70-0.82)^a^ |
| Specificity (95% CI) | 0.66 (0.66-0.67) | 0.66 (0.66-0.67) |
| PPV (95% CI) | 0.03 (0.02-0.03) | 0.02 (0.02-0.02) ^b^ |
| F1-score (95% CI) | 0.05 (0.05-0.05) | 0.04 (0.04-0.04) ^c^ |
| **12-hour** |  |  |
| Sensitivity (95% CI) | 0.93 (0.90-0.97) | 0.79 (0.73-0.84) ^a^ |
| Specificity (95% CI) | 0.73 (0.72-0.73) | 0.69 (0.68-0.69) |
| PPV (95% CI) | 0.03 (0.03-0.03) | 0.02 (0.02-0.02) ^b^ |
| F1-score (95% CI) | 0.06 (0.06-0.06) | 0.05 (0.04-0.05) ^c^ |
| **8-hour** |  |  |
| Sensitivity (95% CI) | 0.93 (0.90-0.97) | 0.80 (0.74-0.85) ^a^ |
| Specificity (95% CI) | 0.73 (0.72-0.73) | 0.70 (0.70-0.71) |
| PPV (95% CI) | 0.03 (0.03-0.03) | 0.02 (0.02-0.03) ^b^ |
| F1-score (95% CI) | 0.06 (0.06-0.06) | 0.05 (0.04-0.05) ^c^ |

The prevalence of ICU transfer with subsequent CDE in the external validation dataset was 0.9%.

The prediction threshold for the F-WIN model was chosen to meet or exceed the specificity of the binarized Bedside PEWS predictions at each prediction horizon. The binarized Bedside PEWS prediction was defined as whether the patient had a Bedside PEWS score greater than 4 during the 24 hours preceding the prediction horizon.

^a^Statistically significant difference (McNemar’s test for comparison of sensitivities, P<0.05) compared with the F-WIN model at the same prediction horizon.

^b^Statistically significant difference (Kosinski’s weighted generalized test for PPV, P<0.05) compared with the F-WIN model at the same prediction horizon.

^c^Statistically significant difference (two-sided permutation test for F1-score, P<0.05) compared with the F-WIN model at the same prediction horizon.

**eTable 12.** Comparison between the F-WIN Model and binarized Clinician Concern prediction on admissions between 2024-07-1 and 2024-12-31.

| **Prediction horizon** | **F-WIN Model** | **Binarized Clinician Concern** |
| --- | --- | --- |
| **24-hour** |  |  |
| Sensitivity (95% CI) | 0.76 (0.63-0.88) | 0.37 (0.22-0.51)^a^ |
| Specificity (95% CI) | 0.92 (0.91-0.93) | 0.92 (0.91-0.93) |
| PPV (95% CI) | 0.08 (0.06-0.09) | 0.04 (0.03-0.06)^b^ |
| F1-score (95% CI) | 0.14 (0.11-0.16) | 0.07 (0.05-0.10) ^c^ |
| **12-hour** |  |  |
| Sensitivity (95% CI) | 0.83 (0.71-0.93) | 0.39 (0.24-0.54) ^a^ |
| Specificity (95% CI) | 0.92 (0.91-0.93) | 0.92 (0.91-0.93) |
| PPV (95% CI) | 0.08 (0.07-0.10) | 0.04 (0.03-0.06) ^b^ |
| F1-score (95% CI) | 0.15 (0.13-0.17) | 0.08 (0.05-0.10) ^c^ |
| **8-hour** |  |  |
| Sensitivity (95% CI) | 0.83 (0.71-0.93) | 0.37 (0.22-0.51) ^a^ |
| Specificity (95% CI) | 0.93 (0.92-0.93) | 0.93 (0.92-0.93) |
| PPV (95% CI) | 0.09 (0.08-0.11) | 0.04 (0.02-0.06) ^b^ |
| F1-score (95% CI) | 0.16 (0.14-0.19) | 0.07 (0.04-0.10) ^c^ |

The prevalence of ICU transfer with subsequent CDE in the external validation dataset was 0.9%.

The prediction threshold for the F-WIN model was chosen to meet or exceed the specificity of the binarized Clinician Concern predictions at each prediction horizon. The binarized Clinician Concern prediction was defined as whether the patient had a Clinician Concern score greater than 0 (i.e. at least slight concern) during the 24 hours preceding the prediction horizon.

^a^Statistically significant difference (McNemar’s test for comparison of sensitivities, P<0.05) compared with the F-WIN model at the same prediction horizon.

^b^Statistically significant difference (Kosinski’s weighted generalized test for PPV, P<0.05) compared with the F-WIN model at the same prediction horizon.

^c^Statistically significant difference (two-sided permutation test for F1-score, P<0.05) compared with the F-WIN model at the same prediction horizon.

# TRIPOD+AI Checklist

# STROBE Checklist

# References

1. Nelson, S.J., et al., *Normalized names for clinical drugs: RxNorm at 6 years.* J Am Med Inform Assoc, 2011. **18**(4): p. 441-8.

2. Carter, J.S., et al., *Categorical information in pharmaceutical terminologies.* AMIA Annu Symp Proc, 2006. **2006**: p. 116-20.
